# Synthetic ditempolphosphatidylcholine liposome-like nanoparticles for anti-oxidative therapy of atherosclerosis

**DOI:** 10.1039/d3ra01822a

**Published:** 2023-05-31

**Authors:** Chunxiao Wang, Ruifu Zhao, Zhen Wang, Tingting Xu, Peng Huang

**Affiliations:** a Department of Cardiology, The Affiliated Yantai Yuhuangding Hospital of Qingdao University Yantai 264000 China huangpengyhd@163.com

## Abstract

Atherosclerosis (AS), a chronic inflammatory disease, is the leading cause of death worldwide. Anti-oxidative therapy has been developed for AS therapy in light of the critical role of ROS in pathogenesis of AS, but current anti-oxidants have exhibited limited outcomes in the clinic. Herein, new ROS-eliminating liposome-like NPs (Tempol-Lips) were assembled from synthetic lipids that covalently conjugated two Tempol molecules with phosphatidylcholine by esterification reaction. The obtained Tempol-Lips can be efficiently internalized into inflammatory macrophages and attenuated inflammation *via* scavenging overproduced intracellular ROS. After i.v. administration, Tempol-Lips with nanoscale character accumulated in the plaques of ApoE^−/−^ mice through passive targeting and significantly inhibited the pathogenesis of AS, compared with those treated with control drugs. The therapeutic benefits of Tempol-Lips primarily are ascribed to the reduced local and systematic oxidative stress and inflammation. Preliminary studies *in vivo* further demonstrated Tempol-Lips were safe and biocompatible after long-term i.v. injection. Conclusively, Tempol-Lips can be developed as a novel anti-AS nanotherapy with potential translation in the clinic.

## Abstract 2

Liposome-like nanoparticle assembly from novel ditempolphosphatidylcholine lipids (Tempol-PC) with ROS-scavenging capability and their therapeutic action on AS.

## Graphical abstract

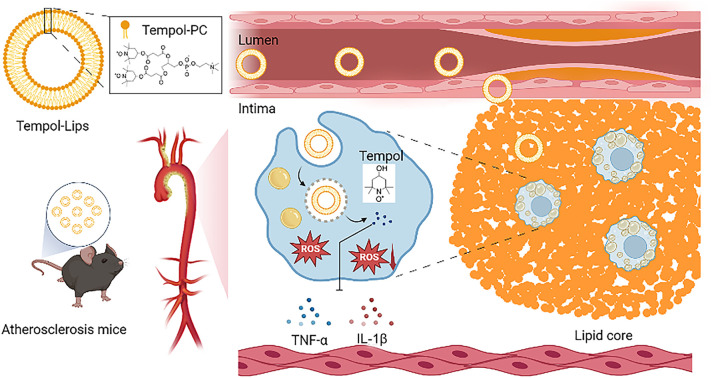

## 1. Introduction

Atherosclerosis (AS) is a chronic inflammatory disease distinguished by lipid and inflammatory cell accumulation in arterial walls.^1–3^ The pathogenesis of AS always involves inflammatory reactions and increased oxidative stress.^4,5^ Oxidative stress is the imbalance in favor of reactive oxygen species (ROS) over-production and/or the body's innate anti-oxidant capability decreases. ROS plays a critical role in inflammation, apoptosis and oxidation of low-density lipoprotein cholesterol (LDL-C), which is closely associated with the pathogenesis of AS.^6,7^ Oxidized LDL (oxLDL) possesses several proatherogenic activities, including foam cell formation,^8^ ROS generation^9^ and adhesion molecule and scavenger receptor expression.^10–12^ Moreover, ROS can also interrupt the redox-dependent pathways in the walls of blood vessels to promote the development of AS, which concerns regulatory genes associated with vascular function, signal transduction pathways and inflammatory components of AS.^5,13,14^ Therefore, reducing ROS generation and attenuating systemic oxidative stress in AS plaques represent reasonable strategies for AS treatment.

Various kinds of anti-oxidants have been studied in preclinical, such as probucol,^15^ vitamins E,^16^ Tempol,^17^ coenzyme Q^18^ and NADPH oxidase.^19^ Although determined with protective effects of anti-oxidants on AS, clinical trials showed no positive effects.^20^ To some extent, this mainly is due to the rapid elimination, nonspecific distribution and low delivery efficiency at targeted plaque of AS *in vivo*.^21^ In addition, the limited ROS-scavenging capability of most single anti-oxidant also contributes to the ineffective outcomes against inflammatory diseases.^22^ Accordingly, additional anti-oxidative stress strategies remain to be designed.

Much evidence demonstrated that nanoparticle (NPs)-based approaches are promising and effective for targeted therapy of AS.^23–26^ NPs can target plaques by direct infiltration or endocytosed by circulating phagocytes, following translocation to AS lesions by cellular infiltration and recruitment. Except that NPs in most studies were just used as vehicles for targeted delivery of therapeutics to AS plaques, recent progress indicated NPs with intrinsic anti-inflammation and anti-oxidative activity are potential next-generation therapy for AS and other inflammatory illness.^27,28^ For instance, NPs assembled from Tempol-containing amphiphilic copolymers were effective to treat drug-induced intestinal inflammation.^29^ A biodegradable polymer-based NPs containing an antioxidant *p*-hydroxybenzyl alcohol can significantly suppressed the inflammation in ischemic tissues of C57BL/6J mice.^30^ However, it should be noted that these developed NPs with complicated polymeric structures are challengeable for reproducible synthesis, structural tailoring and quality control in large-scale production. Besides, *in vivo* clearance performance, degradation, metabolism and safety profile of NPs are still unknown, which facilities the further development of intrinsically active and translational NPs in targeted therapy of AS.

**Scheme 1:**
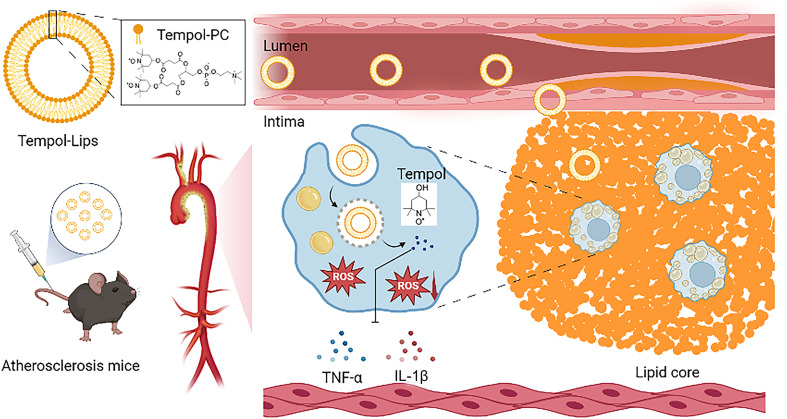
Schematic illustration of Tempol-Lips and *in vivo* antioxidative therapy of atherosclerosis.

Herein, a newly kind of synthetic liposomes-like NPs with ROS-eliminated capability was developed, where the fatty acid chains of typical lipids were replaced by two Tempol molecules. Compared with the studied polymeric NPs assembled from complicated amphiphilic polymers, our work is purely relied on well-defined ditempolphosphatidylcholine (Tempol-PC, Scheme 1). By eliminating ROS, Tempol-PC-based NPs, abbreviated as Tempol-Lips can significantly reduce oxidative stress-induced inflammation in AS lesion. *In vivo* therapeutic investigations in ApoE^−/−^ mice proved that Tempol-Lips inhibited the progression of AS by decreasing local and systemic oxidative stress and inflammation. This strategy provides an efficacious and safe nanotherapy for treatment of AS.

## 2. Materials and methods

4-Hydroxy-2,2,6,6-tetramethylpiperidine-1-oxyl (Tempol, purity ≥ 98%) was obtained from Aladdin Biochemical Technology Co., Ltd (Shanghai, China). l-α-Glycerophosphocholine (GPC, purity ≥ 99%), succinic anhydride (SA, purity ≥ 98%), cholesterol (Chol, purity ≥ 98%), *N*,*N*′-carbonyldiimidazole (CDI, purity ≥ 99%), 4-dimethylaminopyridine (DMAP, purity ≥ 98%) and 1,8-diazabicyclo[5.4.0]undec-7-ene (DBU, purity ≥ 99%) were purchased form DB Technology Co., Ltd (Shanghai, China). RPMI medium 1640, fetal bovine serum (FBS), methyl tetrazolium (MTT), 4′,6-diamidino-2-phenylindole (DAPI) and penicillin–streptomycin solution were supplied by Beyotime (Shanghai, China). Lipopolysaccharide (LPS), 2,2-diphenyl-1-picrylhydrazyl (DPPH) and Oil Red O (ORO) were purchased from Sigma-Aldrich (MO, USA). The chemicals and solvents were provided from the domestic suppliers and used without further purification, except where specified below.

### 2.1 Synthesis of ditempolphosphatidylcholine lipids

**Fig. 1:**
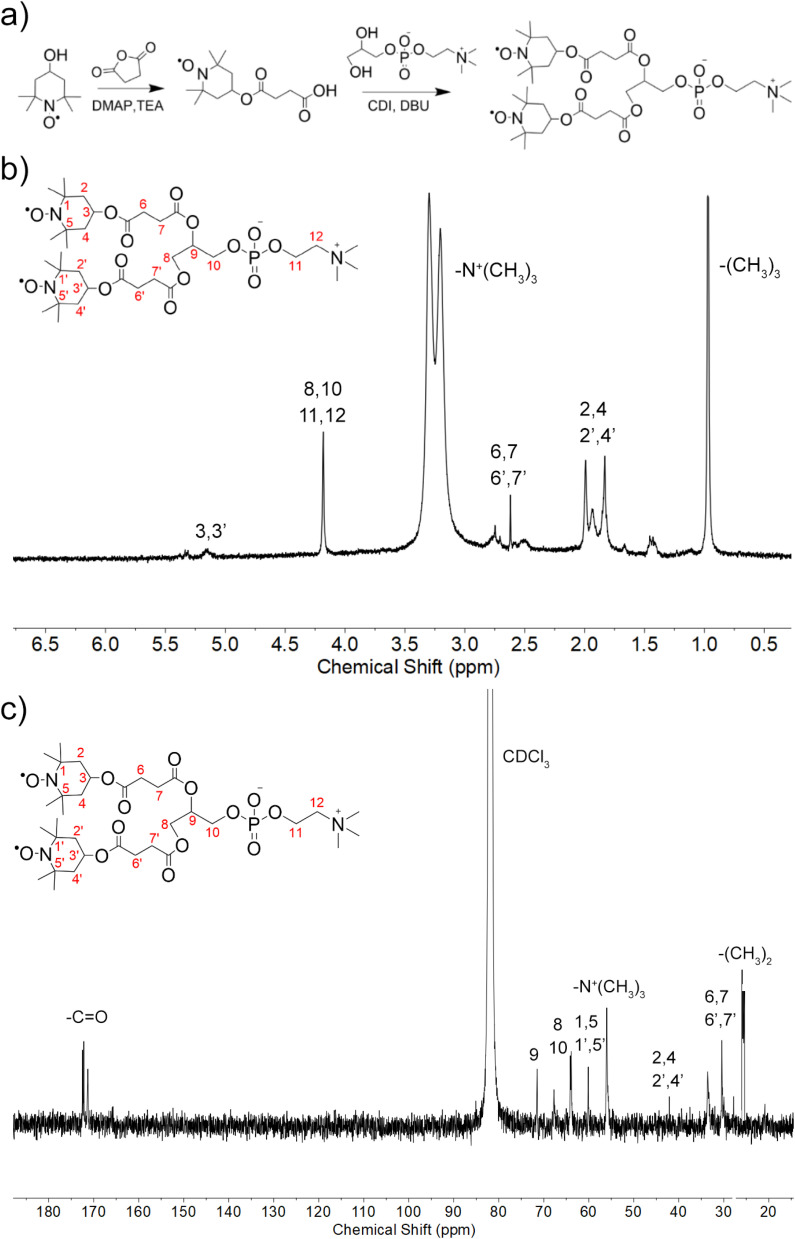
Synthesis and characterization of Tempol-PC lipids. (a) Schematic illustration of synthetic route; (b) ^1^H NMR and (c) ^13^C NMR spectra of Tempol-PC lipids in CDCl_3_.

Lipids ditempolphosphatidylcholine (Tempol-PC lipids) were synthesized through conjugating Tempol onto GPC, as shown in Fig. 1a. Tempol reacted with succinic anhydride (Tempol-COOH) was firstly prepared. Briefly, succinic anhydride (1.76 g/17.43 mmol) was mixed with a solution of Tempol (1 g/5.81 mmol) and DMAP (0.36 g/2.91 mmol) in anhydrous CH_2_Cl_2_ and allowed to react for 0.5 h at 35 °C. After washed with 0.1 M HCl three times, the synthesized product was purified by silica gel column chromatography eluting with ethyl acetate/hexane (2 : 1, v/v) to give Tempol-COOH intermediate as a light pink solid. HRMS *m*/*z*: calculated for C_13_H_22_NO_5_ [M–H]^−^, 271.15; found 271.14 [M–H]^−^, 543.29 [2M–H]^−^.

Next, Tempol-COOH (0.5 g/1.84 mmol) was added to a solution of CDI (0.45 g/2.76 mmol) in dry CH_2_Cl_2_ and reacted for 4 h at room temperature. Without further purification, GPC (0.22 g/0.84 mmol) and DBU (0.28 g/1.84 mmol) dissolved in DMSO were added into the system, heated and stirred at 45 °C overnight. The resulting products were precipitated in diethyl ether (40 mL × 3) acidified with 2 mL of glacial CH_3_COOH and then, purified by silica gel column chromatography using CH_2_Cl_2_/CH_3_OH (solvent A: CH_2_Cl_2_ : CH_3_OH, 15 : 1; solvent B: CH_2_Cl_2_ : CH_3_OH : H_2_O 65 : 25 : 4) as the gradient elution. Finally, Tempol-PC lipids were obtained by evaporating to yield the pink solid of 0.23 g (purity 98.3%). ^1^H-NMR (600 MHz, CDCl_3_): *δ* 5.03, 4.35, 3.25, 2.67, 1.92, 1.68, 0.96 ppm; ^13^C-NMR (600 MHz, CDCl_3_): *δ* 172.64, 72.56, 70.79, 66.07, 64.39, 58.00, 54.38, 43.16, 32.04, 25.75 ppm. HRMS *m*/*z*: calculated for C_34_H_60_N_3_O_14_P [M + H]^+^, 766.38; found 766.38 [M + H]^+^.

### 2.2 Tempol-Lips formulation

Tempol-Lips were prepared by using conventional thin film technique, with some modifications.^31^ An aqueous solution of Tempol-PC lipids (10 mg) in CHCl_3_ was evaporated to form a thin film cling to the walls of flask. This film was hydrated with 5 mL PBS (pH 7.4) solution during rotation for 0.5 h at 50 °C. Liposomes-like NPs were finally collected by homogenization with mini-extruder set (Avanti Polar Lipids Inc., Alabaster, AL) and filtration through 220 nm aseptic membrane, prior to different characterizations. The concentration of Tempol-PC lipids was determined by UPLC (ACQUITY Arc System, Waters, MA, USA).

### 2.3 Particle size and zeta-potential measurements

Hydrodynamic diameter and zeta-potential of Tempol-Lips were measured using dynamic light scattering (DLS) technique equipped with Zetasizer NanoZS90 and zeta-potential analyzer (Malvern Instruments Ltd, Worchestershire, UK). Average particle size and zeta-potential of liposomal formulations were determined in distilled water or serum-containing growth medium.

### 2.4 Morphological characterization

Morphology of Tempol-Lips with a concentration of 200 μg mL^−1^ in PBS (pH 7.4) was analyzed by transmission electron microscopy (TEM). Sample was placed on a carbon film copper grid, air-dried and negatively stained with phosphomolybdic acid (2%, w/v). The images of TEM were visualized by JEM-2100 system (JEOL, Japan), which was operated at an acceleration voltage of 80 kV.

### 2.5 ROS-scavenging capability

ROS-scavenging capability of Tempol-Lips was measured using a previously established protocol.^32^ Briefly, various concentrations of Tempol-Lips ranged from 0 μg mL^−1^ to 200 μg mL^−1^ were incubated with 2 mL of PBS (pH 7.4) containing 500 mM H_2_O_2_ for 48 h. The remaining H_2_O_2_ was quantified by a fluorometric hydrogen peroxide assay kit (Sigma-Aldrich, MO, USA), and eliminated H_2_O_2_ was calculated.

The superoxide anion-scavenging capability of Tempol-Lips was evaluated by incubation with superoxide anion at 37 °C for 40 min, using a commercially available test kit (Beyotime, Shanghai). Moreover, the free radical scavenging capability of Tempol-Lips was further measured. To this end, 1 mL of DPPH˙ (100 μg mL^−1^) was treated with different amount of Tempol-Lips at 37 °C for 0.5 h, following recording the absorbance at 517 nm by UV-visible spectroscopy.

### 2.6 Release kinetics of Tempol-Lips *in vitro*

Appropriate 1 mL of Tempol-Lips (1 mg mL^−1^) in a dialysis tube (MWCO 1000) was incubated with 5 mL of release medium (PBS, pH 7.4 or 5.0) at 37 °C and 100 rpm. At specific time intervals, 0.5 mL of samples were extracted and further mixed with chromatographic grade CH_3_OH of equal volume. The released Tempol amount was determined by UPLC (ACQUITY Arc System, Waters, MA, USA) eluted with CH_3_OH/H_2_O (60/40%, v/v). Flowrate: 1.0 mL min^−1^. Temp.: 25 °C. Detection: 240 nm.

### 2.7 Cell culture

Murine macrophage RAW264.7 cells provided by Cell Bank, Chinese Academy of Science (CAS, Shanghai, China) were cultured in RPMI 1640 media with FBS (10%, v/v), streptomycin (100 mg mL^−1^) and penicillin (100 U mL^−1^) in a humidified environment of 5% CO_2_ in air at 37 °C. Cells were harvested for the following experiments as the pre-cultured cells reached 80% confluence.

### 2.8 Cytotoxicity evaluation

1.0 × 10^4^ cells per well of RAW264.7 cells were cultured with 96-well plates and allowed to attach for overnight. Cells were treated with Tempol-Lips at different doses for predetermined 24 h. The cell viability was quantified by MTT assay per manufacturer instructions and analyzed at 490 nm using a Model 680 Microplate Reader (Bio-Rad, CA, USA). Cells without treatment was used as controls. The percentage of cell viability was presented as comparison with the cells incubated in DMEM medium while tested in sixtuplicates and performed in quartets.

### 2.9 Cellular uptake

RAW264.7 cells were cultured in 3.5 cm-confocal dished containing 2 mL of growth RPMI-1640 medium for 12 h. After treated with 1 μg mL^−1^ LPS in free medium, equivalent 5 μM of Cy5.5-loaded Tempol-Lips was added for further 2 h incubation. RAW264.7 cells were rinsed and stained with DAPI (100 μM in PBS). Finally, confocal laser scanning microscopy (CLSM) was conducted to acquire fluorescence photos.

For flow cytometric analysis, RAW264.7 cells with a density of 1 × 10^6^ cells per well were cultured in 6-well plates for 12 h attachment. Then the cells were treated with 1 μg mL^−1^ LPS and further incubated with 5 μM of Cy5.5-loaded Tempol-Lips. At predefined time points, cells were collected for flow cytometric analysis (BD FACSCanto, NJ, USA).

### 2.10 Anti-inflammatory effects *in vitro*

Briefly, RAW264.7 cells were seeded in 6-well plate at 1 × 10^6^ cells per well. After 12 h, cells were treated with Tempol-Lips or free Tempol for 2 h and then stimulated with 1 μg mL^−1^ LPS for 24 h. The inflammatory cytokines including TNF-α and IL-1β in the culture supernatant were determined by ELISA assays, where the levels of total protein were analyzed by BCA (Beyotime, Shanghai, China).

### 2.11 Intracellular ROS generation

RAW264.7 cells were pretreated with free Tempol and Tempol-Lips (10 μM) for 2 h and stimulated with LPS (1 μg mL^−1^). The normal control group was treated with fresh medium, while model group was only stimulated with LPS at equivalent dosage. Subsequently, cells were incubated with 10 μM of DCFH-DA in serum-free RPMI 1640 for 0.5 h in the dark at 37 °C. The intracellular ROS change was observed by parallel CLSM (FV3000, Olympus, Japan), and the fluorescence intensity of green DCF was determined using FCM (BD FACSCanto, NJ, USA).

### 2.12 Animals

Animals tests and care were performed in consistence with the Provision and General Recommendation of Chinese Experimental Animal Administration Legislation and approved by the Institutional Animal Care and Use Committee of the Affiliated Yantai Yuhuangding Hospital of Qingdao University. Male ApoE^−/−^ mice (6–8 week) were supplied by the Pengyue Pharmaceutical Co., Ltd (Jinan, China), preserved in a humidity standard (60 ± 5%) and controlled temperature (22 ± 2 °C) animal room and allowed free access to food and water.

### 2.13 Pharmacokinetic and plaque targeting study

The pharmacokinetic profile of Tempol-Lips was studied using Cy5.5-loaded liposomes after i.v. injection. Cy5.5-loaded Tempol-Lips was administrated to ApoE^−/−^ mice at 1.2 μg kg^−1^. At predefined time points, 100 μL of whole blood samples was obtained in 96-well black plate. For plaque targeting investigation, male ApoE^−/−^ mice administrated with Cy5.5-loaded Tempol-Lips were perfused with 4% paraformaldehyde, euthanized, and the aortas were isolated. *Ex vivo* imaging was observed by an IVIS spectrum system (Lumina 3, PerkinElmer, CA).

### 2.14 Therapeutic efficiency

Fifty ApoE^−/−^ mice after fed with high-fat diet for 2 months were randomly assigned into 5 groups (*n* = 10). Then mice were treated with 0.9% saline, probucol (5.2 mg kg^−1^), free Tempol (17.2 mg kg^−1^) and Tempol-Lips (20 mg kg^−1^). Probucol was dissolved in saline with 30% ethanol and free Tempol was in saline. All formulations were i.v. injected every other day for continuous 1 month.

### 2.15 ORO staining

After various treatments, ApoE^−/−^ mice were sacrificed. The aorta was resected and then perfused with 10% formalin for 1 h. The aorta was opened longitudinally and stained with ORO. Moreover, cryo-sections of aortic root was further stained with ORO. Plaque area analysis was carried out with the Image-Pro Plus 6.0 software.

### 2.16 Dihydroethidium staining

Aortic roots of different groups were embedded in Tissue-Tek O. C. T. compound, and 6 μm-thickness sections were achieved on a Leica cryostat. Dihydroethidium (DHE) as fluorescent dye was used to probe the generation of superoxide anion *in situ*, according to description reported previously. The sections were incubated with 2% triton X-100 and blocked with 5% BSA in PBS. Afterwards, slides were stained with DHE (2 μM) in Krebs solution and finally observed by CLSM.

### 2.17 Inflammatory cytokines determination

The excised aortas were homogenized and centrifuged in saline. Supernatant samples were collected and the levels of TNF-α and IL-1β inflammatory cytokines were measured using ELISA assays (Beyotime, Shanghai, China). Similarly, TNF-α and IL-1β cytokines in serum were determined.

### 2.18 Serum lipids measurements

After different treatments, the levels of triglycerides (TG), total cholesterol (TC), low-density lipoprotein cholesterol (LDL-C) and high-density lipoprotein cholesterol (HDL-C) in serum were measured using commercially available kits (Solarbio, Beijing, China).

### 2.19 Safety evaluation

During the treatment period, the body weight of ApoE^−/−^ mice were weighted every other week for possible adverse effects. Aortas and major tissues were further dissected and analyzed for H&E staining, as described previously.

### 2.20 Statistical analysis

Results were expressed as mean ± SD, and statistical analysis between groups was analyzed by GraphPad Prism Software (Version 8.0, La Jolla, CA, USA) using one-way ANOVA. Statistical significance was assessed at *P < 0.05 and **P < 0.01.

## 3. Results and discussion

### 3.1 Synthesis, preparation and characterization of Tempol-Lips

Amphiphilic Tempol-PC lipids was synthesized by a two-step reaction, according to the scheme in Fig. 1a. As shown, Tempol-COOH intermediate was first prepared by esterification of Tempol and SA under the catalytic condition of DMAP and TEA, following covalently conjugated to GPC. The synthesized Tempol-PC lipids were successfully by ^1^H NMR and ^13^C NMR spectra (Fig. 1b and c). Compared with ^1^H NMR spectrum of the intermediate Tempol-COOH, newly appeared peaks at 3.25 ppm is belong to the methyl protons (–N^+^(CH_3_)_3_) from GPC molecule. In addition, the peaks at 54.3 ppm are the characteristic carbon signals of –N^+^(CH_3_)_3_ in ^13^C NMR spectrum, which further confirmed the esterification between Tempol-COOH and GPC.

**Fig. 2:**
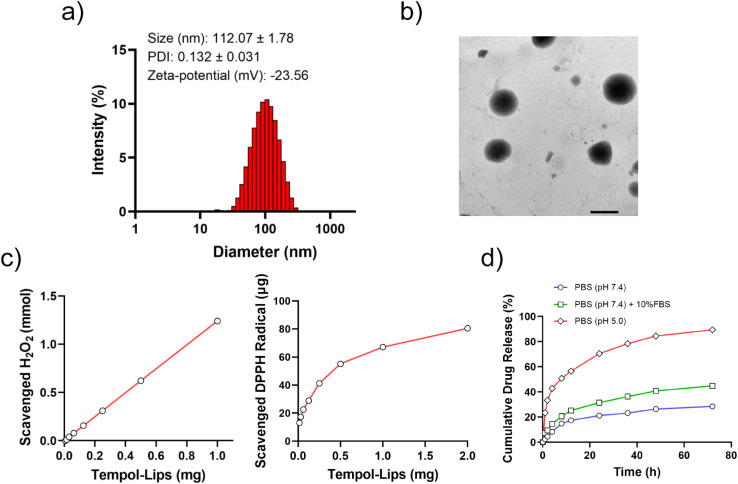
Characterization and ROS-scavenging capability of Tempol-Lips. (a) Size distribution and zeta-potential profile of Tempol-Lips and (b) representative TEM image. Scale bar = 200 nm; (c) dose-dependent scavenge of H_2_O_2_ and DPPH radical by Tempol-Lips; (d) release kinetics of Tempol-Lips in PBS with different pHs at 37 °C.

Liposomes-like NPs (Tempol-Lips) assembled from Tempol-PC lipids were prepared by a conventional thin film technique. The average size and morphology of Tempol-Lips were studied by using DLS and TEM. As displayed in Fig. 2a, the hydrodynamic diameter of Tempol-Lips was 112.07 ± 1.78 nm with a negatively charged zeta-potential of −23.56 mV in PBS (pH 7.4), indicating the formation of NPs with a narrow size distribution (PDI = 0.132). Characterization by TEM revealed the spherical morphology for the desired Tempol-Lips (Fig. 2b). These results collectively demonstrated the self-assembly of Tempol-PC lipids, while the dual Tempol molecules play a critical role in the assembly of Tempol-Lips. Based on the well-defined structure of Tempol-PC, the content of Tempol loaded in NPs is higher upto 44.9% calculated by molecular weight after a simple calculation.

Tempol is a free radical scavenger with equivalent efficacity of superoxide dismutase (SOD), which has been demonstrated effective in treatment of oxidative-stress-related diseases.^17,33^ Thus, *in vitro* ROS-eliminating capability of assembled Tempol-Lips was studied and results were shown in Fig. 2c. As expected, Tempol-Lips scavenged H_2_O_2_ in a dose-dependent manner after 48 h post-incubation. Similarly, it was also found that the eliminated DPPH radical was remarkably increased along with the improved concentration of Tempol-Lips, confirming their significant ROS-scavenging ability. These results potentiated Tempol-Lips as anti-AS platform in *in vivo* administration. Moreover, different simulated pHs (7.4 or 5.0) with 10% FBS or not were used to investigate the kinetic release of Tempol from Tempol-Lis by dialysis (MWCO 1000) method at 37 °C (Fig. 2d). Appropriately 40.5% of parent Tempol was released form Tempol-Lips after 72 h of incubation in PBS (pH 7.4) solution with FBS (10%, v/v), whereas Tempol-Lips showed an accelerated dissociation and acid-responsive Tempol release upto 92.1% at pH 5.0. Accordingly, the breakage of ester bonds incorporated in Tempol-Lips at low pH would lead to the disintegration and payload release under acidic microenvironment of inflammatory macrophages in AS lesions,^34,35^ therefore contributing their possibilities of enhancing anti-AS activity *in vivo*.

### 3.2 Anti-oxidation and anti-inflammation of Tempol-Lips *in vitro*

**Fig. 3:**
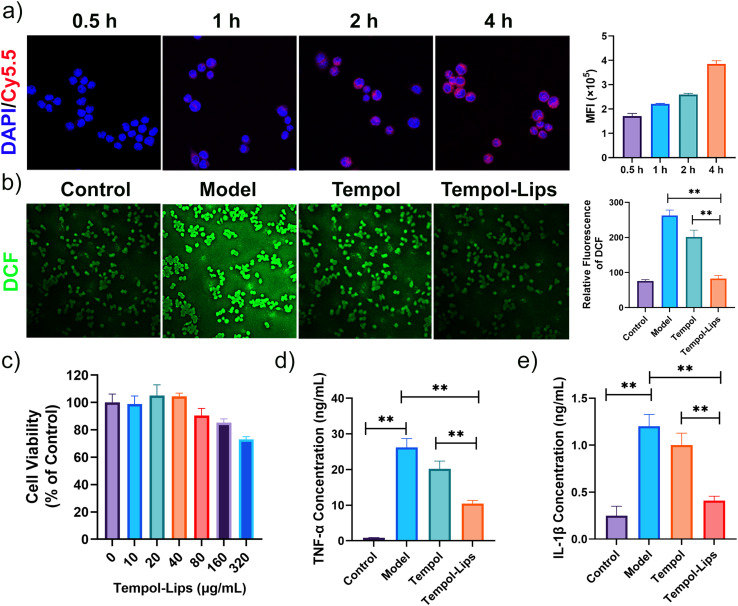
Cellular uptake of Tempol-Lips and their biological effects against LPS-activated RAW264.7 cells. (a) CLSM images and quantification of time-dependent internalization of Cy5.5-loaded Tempol-Lips. Nuclei were stained with DAPI (blue). Scale bar = 20 μm; (b) intracellular ROS generation in RAW264.7 cells treated with free Tempol and Tempol-Lips, followed by stimulation with LPS for 4 h determined by CLSM and FCM, respectively. Scale bar = 40 μm; (c) cell viability of RAW264.7 cells *in vitro* after incubation with various dosed of Tempol-Lips for 24 h; expression of (d) TNF-α and (e) IL-1β inflammatory cytokines by LPS-activated RAW264.7 cells. Cells were preincubated with different concentration of Tempol-Lips for 2 h and then stimulated by LPS for 24 h. The levels of cytokines were analyzed by ELISA. Data were shown as mean ± SD. Statistical significance was assessed at *P < 0.05 and **P < 0.01.

Overproduced ROS and sustained oxidative stress can induce cell and tissue damage, which in turn triggers inflammatory circulation and results in amplification of oxidative stress.^36^ The anti-oxidation of Tempol-Lips was checked against inflammatory RAW264.7 macrophages. The model group that RAW264.7 cells were merely stimulated by LPS for 4 h displayed a considerably high level of ROS, as probed by a DCF-DA fluorescent dye with green fluorescence (Fig. 3a). By contract, after pretreated with free Tempol and Tempol-Lips for 2 h, the intensity of fluorescent signals of DCFH-DA was largely reduced, particularly in the case of Tempol-Lips-treated cells. Further quantitative analysis by flow cytometry also revealed that intracellular ROS generation in LPS-activated RAW264.7 cells was maximally inhibited by Tempol-Lips. This promising anti-oxidation of Tempol-Lips can be attributed to the nanostructure of cell membrane-like NPs that is benefit to the enhanced uptake in a manner through adsorption-mediated endocytosis (Fig. 3b) and as a result, more Tempol accumulated into these cells, thereby endowing its anti-inflammation. To investigate this hypothesis, the cytotoxicity of Tempol-Lips was evaluated against RAW264.7 cells using MTT assay (Fig. 3c). After incubation for 24 h, RAW264.7 cells showed >80% cell viability ranged from 10 μg mL^−1^ to 320 μg mL^−1^, disclosing good cytocompatibility of Tempol-PC lipids. Based on the above-mentioned feature, *in vitro* anti-inflammation of Tempol-Lips was finally determined by checking their inflammatory response in macrophages (Fig. 3d and e). Treatment of RAW264.7 cells with LPS stimulation promoted the production of TNF-α and IL-1β pro-inflammatory cytokines, whereas cells pretreated with free Tempol or Tempol-Lips at equivalent dose of 10 μg mL^−1^ were found with inhibitive expression of these cytokines. In particulate, treatment with same doses of Tempol-Lips exhibited more positive on anti-inflammatory effects, compared to that of free Tempol, which is in consistence with the results of enhanced internalization *in vitro*. Previous findings have shown that ROS can activate multiple signal transduction cascades, which in turn modulate inflammation in atherosclerosis.^4,9^ Consequently, Tempol-Lips can attenuate inflammation in macrophages by decreasing intracellular ROS production.

### 3.3 Therapeutic efficacy

**Fig. 4:**
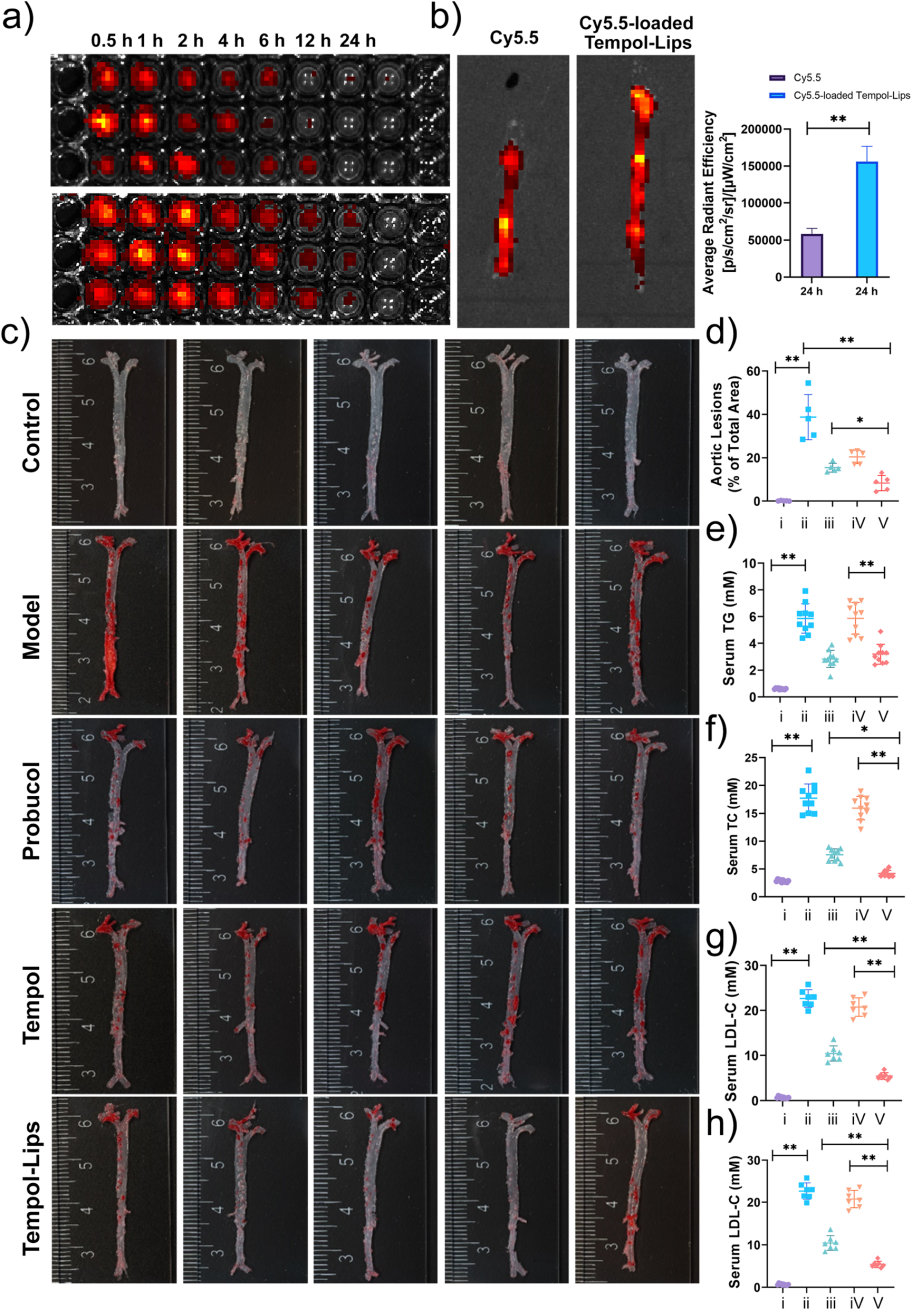
Blood retention and targeting ability *in vivo* of Tempol-Lips after i.v. administration. (a) Pharmacokinetic performance of Cy5.5-loaded Tempol-Lips in ApoE^−/−^ mice (*n* = 3); (b) *ex vivo* fluorescence photos and targeting quantitation of Cy5.5 fluorescence in aortas. At 24 h after single i.v. administration, aortas of ApoE^−/−^ mice treated with Cy5.5-loaded Tempol-Lips were collected for *ex vivo* imaging; (c) representative images of ORO-stained aortas from ApoE^−/−^ after i.v.-delivered Tempol-Lips (*n* = 10); (d) quantitative evaluations of the lesion area in aortas; (e–h) serum levels of TG (e), TC (f), LDL-C (g) and HDL-C (h). (i) control; (ii) model; (iii) probucol; (iv) Tempol; (v) Tempol-Lips. Data were shown as mean ± SD. Statistical significance was assessed at *P < 0.05 and **P < 0.01.

*In vivo* pharmacokinetic profile of Cy5.5-loaded Tempol-Lips was first analyzed in atherosclerotic ApoE^−/−^ mice after 2 month fed with high-fat diet. Following i.v. administration of free Cy5.5 and Cy5.5-loaded Tempol-Lips, blood samples from each group were collected and evaluated by measuring the relative fluorescence intensity at various time points using an *in vivo* fluorescence imaging system (IVIS, PerkinElmer, MA). As shown in Fig. 4a, fluorescence imaging indicated that Tempol-Lips significantly extended blood circulation with a span of 24 h, while free Cy5.5 was almost completely removed from the blood. This phenomenon would be benefit for the accumulation of Tempol in AS plaques. Then the fluorescence in the isolated entire aortas from each group was further recorded to evaluate *in vivo* targeting capability of i.v. injected Tempol-Lips. At 24 h, more significant accumulation of Cy5.5-loaded Tempol-Lips with almost 3-fold higher than free Cy5.5 group was observed (Fig. 4b), suggested that an i.v.-injected Tempol-Lips was able to accumulate in AS lesions.

Together with above potential results, therapeutic efficacy of Tempol-Lips *in vivo* was assessed. After receiving a high-fat diet for 2 months, ApoE^−/−^ mice assigned randomly into 5 groups that were i.v.-treated with different formulations every other day. Apart from free Tempol, probucol, which was demonstrated to be effective as a small-molecule anti-oxidative drug for AS treatment in different animal models, was also used as positive group. At the end of the experiment, the entire aortas were obtained and stained with ORO (Fig. 4c). The saline group with high ORO positive area was seen, clearly indicating the form of atherosclerotic plaques. Treatment with Tempol-Lips afford considerable effects on the decrease of inflammatory plaque at 20 mg kg^−1^ and quantification analysis of the average plaque area was down to 8.35% of total aorta tissue, while the data was 20.42% and 15.38% for mice treated with free Tempol and probucol, respectively. Much better outcomes of Tempol-Lips were significantly achieved to attenuate the development of atherosclerosis. Consistent with this result, analysis on serum levels of TC, TG, LDL-C and HDL-C also revealed the most significant anti-AS activity for Tempol-Lips (Fig. 4e–h). This might be ascribed to their favorable pharmacokinetic profile and targeting capabilities *in vivo*.

**Fig. 5:**
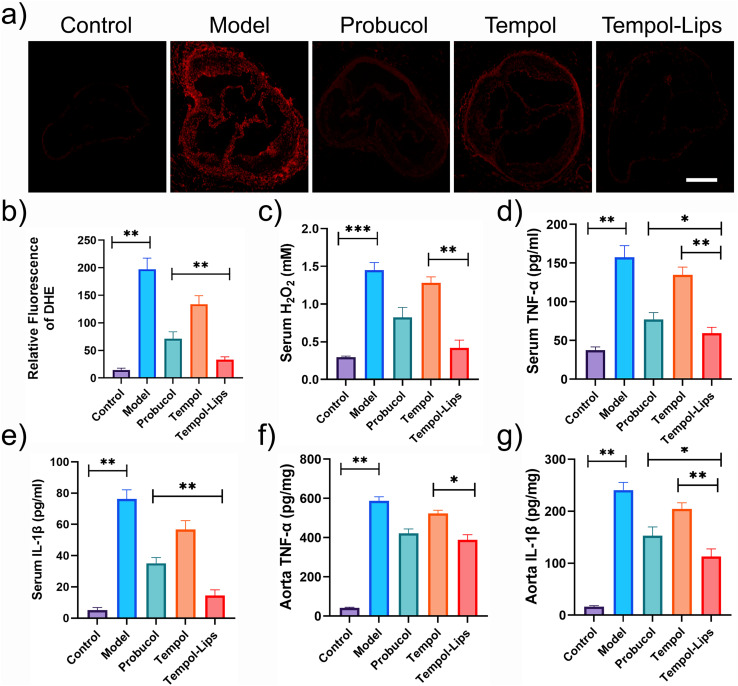
Suppression of oxidative stress and inflammation of Tempol-Lips in ApoE^−/−^ mice. (a) Immunofluorescence images and (b) quantitative analysis of DHE-stained sections of aortic roots. Scale bar = 200 μm; (c) serum levels of H_2_O_2_ and (d–g) levels of TNF-α and IL-1β in serum (d and e) and aortic tissues (f and g) of ApoE^−/−^ mice after treated with different formulations (*n* = 5). Data were shown as mean ± SD. Statistical significance was assessed at *P < 0.05 and **P < 0.01.

The mechanism responsible for *in vivo* anti-AS activity of Tempol-Lips was preliminarily studied by staining aortic root with DHE. As shown in Fig. 5a, sections of aortic roots from saline-treated ApoE^−/−^ were observed with obvious fluorescence, due to the reaction of DHE and superoxide anions that yields ethidium with red fluorescence.^37^ However, observation and quantitative analysis discovered that oxidative stress was drastically suppressed after i.v-treated Tempol-Lips (Fig. 5b). Combined with similar result that lowest levels of ROS were tested (Fig. 5c), it can be concluded both lesional and systematic oxidative stress was mitigated by i.v.-delivered Tempol-Lips. Furthermore, two typical inflammatory cytokines of TNF-α and IL-1β in the serum and aorta from ApoE^−/−^ mice received Tempol-Lips treatment was found with lowest expression, compared with free Tempol and protocol (Fig. 5d–g). Significantly, Tempol-Lips were able to reduce systematic oxidative stress and inflammation effectively as well as decrease inflammation and oxidative stress in AS plaques.

### 3.4 Safety evaluation

**Fig. 6:**
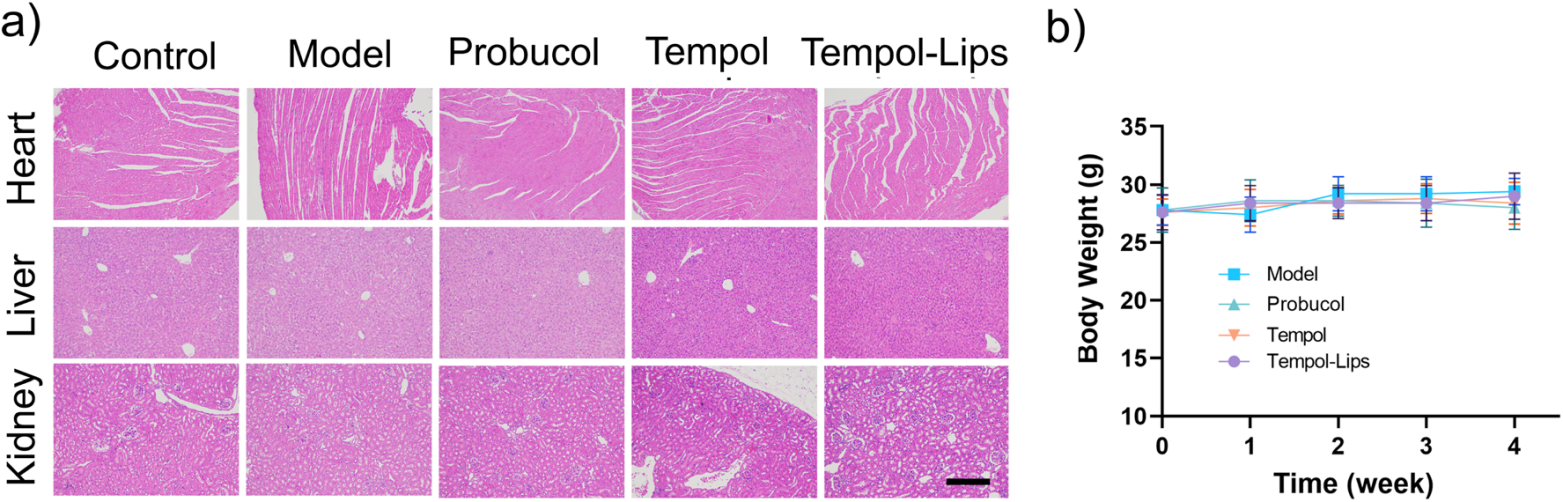
Safety profile during and after treatment with Tempol-Lips. (a) Body weight of ApoE^−/−^ mice in each group studied in therapeutic efficacy; (b) H&E stained sections of major organs. All micrographs were acquired at ×10 magnification. Scale bars = 100 μm.

The possible toxic side effects of NPs are a great matter of safety concern for biological applications in clinic. To evaluate biosafety *in vivo*, H&E-stained histological sections of the major organs (heart, liver, and kidney) were performed on AS model mice after 1 month treatment with different Tempol formulations. No distinct pharmacological lesions were detected in sections of all treated groups (Fig. 6a). In addition, as shown in Fig. 6b, these formulations had little effect on the body weight changes in AS mice model during drug administration, collectively supporting the acceptable biocompatibility of Tempol-Lips at the examined dose.

## 4. Conclusions

A novel kind of ditempolphosphatidylcholine lipids (Tempol-PC) with ROS-scavenging capability was successfully synthesized by replacing fatty acid chains of typical lipids with two Tempol. Through a well-established thin film evaporation approach, Tempol-PC lipids can assemble into a nanotherapy of liposomes-like NPs (Tempol-Lips) that efficiently internalized and eliminated overproduced intracellular ROS in inflammatory macrophages. Importantly, treatment with Tempol-Lips resulted in significant therapeutic outcomes with pathogenesis inhibition of AS compared with those treated with control groups., which was realized by reducing local and systematic oxidative stress and inflammation in plaques. Combined with the safe profile after i.v. long-term administration, Tempol-Lips is efficacious and promising as new nanotherapy for AS therapy.

## Conflicts of interest

The authors declare no conflict of interest.

## Supplementary Material
